# GENetic and clinical Predictors Of treatment response in Depression: the GenPod randomised trial protocol

**DOI:** 10.1186/1745-6215-9-29

**Published:** 2008-05-22

**Authors:** Laura Thomas, Jean Mulligan, Victoria Mason, Debbie Tallon, Nicola Wiles, Philip Cowen, David Nutt, Michael O'Donovan, Deborah Sharp, Tim Peters, Glyn Lewis

**Affiliations:** 1 Department of Community Based Medicine, University of Bristol, 25 Belgrave Road, Bristol, BS8 2AA, UK; 2 Psychology and Health Sciences, University of Worcester, Henwick Grove, Worcester, WR2 6AJ, UK; 3 University Department of Psychiatry, Neurosciences Building, Warneford, Hospital, Oxford, OX3 7JX, UK; 4 Department of Psychological Medicine, University of Wales College of Medicine, Heath Park, Cardiff, CF14 4XN, UK

## Abstract

**Background:**

The most effective pharmacological treatments for depression inhibit the transporters that reuptake serotonin (Selective Serotonin Reuptake Inhibitors – SSRIs) and noradrenaline (Noradrenaline Reuptake Inhibitors – NaRIs) into the presynaptic terminal. There is evidence to suggest that noradrenaline and serotonin enhancing drugs work through separate mechanisms to produce their clinical antidepressant action. Although most of the current evidence suggests there is little difference in overall efficacy between SSRIs and NaRIs, there are patients who respond to one class of compounds and not another. This suggests that treatment response could be predicted by genetic and/or clinical characteristics.

Firstly, this study aims to investigate the influence of a polymorphism (SLC6A4) in the 5HT transporter in altering response to SSRI medication. Secondly, the study will investigate whether those with more severe depression have a better response to NaRIs than SSRIs.

**Methods/design:**

The GenPod trial is a multi-centre randomised controlled trial. GPs referred patients aged between 18–74 years presenting with a new episode of depression, who did not have any medical contraindications to antidepressant medication and who had no history of psychosis or alcohol/substance abuse. Patients were interviewed to ascertain their suitability for the study. Eligible participants (with a primary diagnosis of depression according to ICD10 criteria and a Beck Depression Inventory (BDI) score > 14) were randomised to receive one of two antidepressant treatments, either the SSRI Citalopram or the NaRI Reboxetine, stratified according to severity. The final number randomised to the trial was 601. Follow-up assessments took place at 2, 6 and 12 weeks following randomisation. Primary outcome was measured at 6 weeks by the BDI. Outcomes will be analysed on an intention-to-treat basis and will use multiple regression models to compare treatments.

**Discussion:**

The results of the trial will provide information about targeting antidepressant treatment for individual patients; in turn this may increase prescribing efficacy, thereby speeding recovery and reducing the cost to the NHS. It will also help to understand the different roles that noradrenaline and serotonin might play in the biology of depression.

The trial is expected to report in the autumn of 2008.

**Trial Registration:**

ISRCTN 31345163

## Background

Depression is the most common mental disorder in community settings. Indeed, the Global Burden of Disease study suggests that depression will be second only to cardiovascular disease in causing disability by the year 2020[1].

Depression is an illness that is characterised by low mood and the inability to experience pleasure. Symptoms can impact upon the emotional, cognitive, physical and behavioural health of the individual, although not every patient experiences all symptoms. Behavioural and physical symptoms can include irritability, tearfulness, reduced sleep, increased pain (with new or existing symptoms), reduced appetite, fatigue, loss of interest in everyday life, guilt and worthlessness. Cognitive symptoms include poor concentration, forgetfulness, pessimism, negative thoughts about the past and future, and slower mental aptitude.

In the UK depression is usually treated in primary care and in 2005 there were in excess of 29 million prescriptions for antidepressant medication[2]. The cost of treatment for depression in the National Health Service (£887 million) is greater than both that for hypertension and diabetes combined (£439 and £300 million respectively)[3]. However, these figures do not take account of the indirect costs associated with depression[4], and it is also important to consider the wider social implications of depression beyond that pertaining directly to the individual. Social and occupational functioning is limited when an individual is depressed, with consequently increased dependence upon welfare and benefits. In particular, days lost from work due to depression exceed all other disorders. In 1994 an estimated 1.5 million disability-adjusted life years were lost each year in the developed world as the result of depression[5]. Finding an effective treatment for depression is therefore a key consideration for the health service.

The most effective and widely used pharmacological treatments for depression inhibit the transporters that reuptake serotonin (5-hydroxytryptamine -5HT) and noradrenaline (NA) into the presynaptic terminal. They demonstrate a range of affinities, from selectivity for 5HT (SSRIs), via compounds with affinity for both NA and 5HT, to those that are selective for NA. Specific noradrenaline reuptake inhibitors (NaRIs), such as Reboxetine, have recently been developed. National Institute for Health and Clinical Excellence (NICE) guidelines in the UK recommend SSRIs as first-line pharmacological treatment for moderate and severe depression. While SSRIs are effective in many cases there are some patients who do not respond to this medication. It has been reported however, that some dual-acting drugs such as serotonin-noradrenaline reuptake inhibitors (SNRIs), or tricyclic antidepressants such as amitriptyline, are more efficacious when treating patients to remission than SSRIs[6,7]. This research suggests it is important to consider the utility of enhancing noradrenaline activity in some patients rather than focusing solely on increasing serotonin function. Perhaps alternatives to SSRIs such as NaRIs may also help in these cases.

Interactions between NA and 5HT pathways make it likely that NA- and 5HT-potentiating drugs have some pharmacological actions in common, particularly after longer-term use; however, there is now good evidence for specificity of pharmacological action. Depressed patients who have responded clinically to SSRIs relapse when 5HT but not NA neurotransmission is interrupted. Exactly the converse has been observed with patients who have responded to NA-potentiating drugs such as desipramine[8]. This suggests that NA and 5HT-enhancing drugs work through separate mechanisms to produce their clinical antidepressant action, and the development and promotion of new NaRIs is expected to occur.

Most of the current evidence suggests that there is little difference in overall efficacy between SSRIs and NaRIs[9]. Nevertheless clinical experience indicates that some patients respond to one class of compounds and not to the other, suggesting that treatment response could be predicted by genetic and/or clinical characteristics. There is, however, a dearth of reliable evidence in the literature regarding specific predictors of (differential) response to these two classes of medication. Previous investigations, conducted primarily within the pharmaceutical industry, have deficiencies such as not being available in the peer-reviewed literature, lack of power, unclear prior hypotheses and multiple hypotheses testing.

In summary, there is currently a 30–40% chance that a patient will not recover with a first antidepressant. If they fail to respond to this first type of medication, the likelihood that they will not respond to the second is about 50%. A method of targeting medication for an individual based on genetic or clinical characteristics would improve these statistics and enable GPs to prescribe more effectively.

## Aim

The aim of this paper is to describe the protocol for a randomised controlled trial (RCT) designed to investigate genetic and clinical predictors of treatment response to SSRIs and NaRIs in depressive illness. There are two hypotheses concerned with response to NaRIs and SSRIs.

### 1. Polymorphism in the serotonin transporter

The most interesting polymorphism in the gene that encodes the serotonin transporter (SLC6A4) is a 44 bp-insertion/deletion polymorphism within a repetitive unit in the 'promoter' region. Both reporter gene analyses in cell culture and analysis of native receptors in lymphoblastoid cell lines suggest that the long (insertion) form is functionally more active than the short (deletion) form [10,11]. Human subjects who are homozygotes for the insertion allele (long/long) have a greater prolactin response to fenfluramine when on SSRIs [12]. This has led to the postulation that SSRIs would induce a greater anti-depressant response in individuals who are homozygous for the insertion allele – a hypothesis that has recently been supported by two studies[13,14]. A third study from Korea yielded a 'negative' finding[15], with more recent studies reporting that the findings are inconsistent[16]. All these studies, however, failed to include a comparison group to determine whether the insertion allele influenced prognosis independent of any antidepressant action.

#### Hypothesis 1

Those who are homozygous for the insertion allele polymorphism in the promoter region of the 5HT transporter (SLC6A4) who are allocated SSRIs will have an improved response compared to those on NaRIs. This also implies that those who are not homozygous will have reduced response on SSRIs compared to those on NaRIs.

### 2. Severity of illness

There has been anecdotal clinical evidence that those with more severe depression are more likely to respond to antidepressants with NaRI properties. More formally, two studies have suggested that NaRI is more effective in the more severe depressions[17,18]. It should be noted that severity of depression overlaps with the presence of 'endogenous' or 'biological' symptoms, which in the past were used as clinical indications for antidepressant use. For simplicity, the hypothesis has been formulated here in terms of severity of illness.

#### Hypothesis 2

Those who have more severe depressive disorders who are allocated NaRIs and those who have less severe disorders allocated SSRIs will have a better response compared to the other two groups of patients.

## Methods/design

### Recruitment of participants and baseline assessment

**Figure 1:**
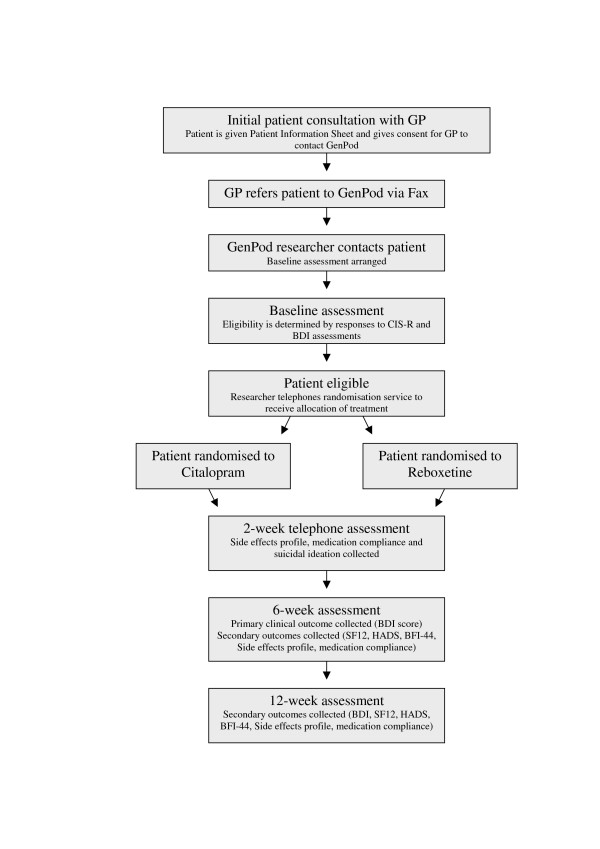
A flowchart of the GenPod trial design.

The study is a multi-centred randomised controlled trial (shown in figure 1) in which patients with depression, recruited in primary care, were randomly allocated to either an NaRI (Reboxetine) or an SSRI (Citalopram). The study includes those aged between 18–74 with the relatively more severe episodes of depression in whom the GP and patient had already agreed that antidepressants should be prescribed. The study is being conducted in three centres within the UK; Bristol, Birmingham and Newcastle. In accordance with the Declaration of Helsinki full ethical approval has been obtained from the South West Ethical Board as well as research governance approval from the Bristol, Manchester and Newcastle PCTs.

The study excluded potential participants who have taken antidepressant medication within two weeks leading up to the baseline assessment and those who could not complete self-administered scales. Moreover, GPs excluded anyone who had medical contraindications or in whom participation in the trial was not deemed appropriate, as well as those who had psychosis, bipolar disorder or major substance or alcohol abuse.

Having secured their written agreement to be contacted by the GenPod research study team, GPs referred suitable patients to the research team by fax, and researchers then contacted the patients directly, inviting them to participate in the study. The researchers visited patients either at home or at the patients' own surgery to assess eligibility and to obtain informed consent for participation within the trial. At the baseline assessment only those patients with a diagnosis of ICD-10 depressive episode F32 from the Clinical Interview Schedule-Revised (CIS-R)[19] and a Beck Depression Inventory (BDI)[20] score of ≥ 15, were eligible to continue in the study. The final number randomised in the trial was 601 subjects.

The CIS-R is a fully structured interview that measures neurotic psychopathology and was designed to detect ICD-10 neurotic disorders. The survey asks questions about the presence of 14 symptom groups in the week prior to interview, with questions to identify the onset and duration of each episode. The BDI is a 21-item self report rating inventory constructed to measure attitudes, severity and symptoms of depression (consistent with DSM-IV criteria), as well as the efficacy of antidepressants. It is not intended as an instrument for specifying clinical diagnosis.

Further measures were collected from eligible patients at baseline, including the SF-12 health questionnaire[21], the Hospital Anxiety and Depression Scale (HADS)[22], a measure of physical symptoms using a modified version of the Toronto Side Effects Scale[23], and the Big Five Inventory personality scale (BFI-44)[24].

### Randomisation procedure

Once patients have met the eligibility criteria for both of these measures they were then randomised to receive one of the two antidepressant medications described above. Randomisation was conducted by means of a computer-generated code, administered centrally and communicated by telephone and thereby concealed in advance from the research assistant. The assigned medication was given to the participant by the research assistant – effectively prescribed by the lead GenPod clinician in the recruiting centre with the agreement of the patient's GP. Overall clinical responsibility for the patient remained with the GP, who was informed of the allocation.

To ensure reasonable balance between the two treatment groups over time in respect of severity, which is the most important prognostic indicator[19], allocation was stratified by severity of depressive episode (CIS-R total score ≥ 28 is classed as severe, below this is classed as moderate), and also stratified by centre using variable block sizes to maximise concealment.

### DNA Sampling

At the baseline assessment, blood samples were also taken from the eligible patients in order to investigate the potential links with the SLC6A4 gene. DNA will be extracted and banked by the Cardiff MRC Co-operative Group in NeuroPsychiatric Genetics, a group with extensive experience of banking DNA under MRC guidelines. Stock DNA will be stored at -20 in a linked anonymised format, with codes held by the Principal Investigator (GL). 27 ml of blood will be taken at the initial assessment and is expected to yield an average of 800 μg.

### Interventions

Both interventions are medicinal. Trial participants were randomised to receive either 4 mg of reboxetine twice daily or 20 mg of citalopram daily. Both medications are believed to be equally effective. The patient was given 6 weeks medication at baseline and the remainder at the 6-week follow-up appointment.

#### (i) Citalopram

Citalopram (brand name *Cipramil*) is a selective serotonin reuptake inhibitor (SSRI) that is licensed to treat depression and also to treat panic disorder. It works by inhibiting the reuptake of the neurotransmitter serotonin from its site of action at the synaptic cleft. By doing so citalopram may prolong the actions of serotonin and this is likely to be the mechanism that underlies its therapeutic action.

SSRIs are often preferred to the tricyclic antidepressants (TCAs) because the side effects of SSRIs are generally better tolerated. SSRIs are also safer in overdose for patients who are highly suicidal.

#### (ii) Reboxetine

Reboxetine (brand name *Edronax*) is a noradrenaline reuptake inhibitor (NaRI) licensed as an antidepressant. Low noradrenaline activity in the brain is believed to be one possible cause of depression. Reboxetine works by inhibiting the re-uptake of noradrenaline, and therefore restoring the levels of noradrenaline in the body. It is also believed to help relieve some of the associated symptoms of depression such as fatigue and anxiety.

Reboxetine is the first NaRI to be marketed and therefore relatively little is known about its tolerance in comparison with other more established types of antidepressants. The use of reboxetine in the current study will provide data on the side effect profile of this medication within this primary care population.

### Follow-up

Clinical outcome data were recorded 6 and 12 weeks after randomisation. However, since side effects are most commonly experienced in the first two weeks of treatment, an additional telephone follow-up was administered after 2 weeks to assess side effects, suicidal ideation and adherence with medication. The latter will be used to identify individuals to be included in the explanatory secondary analyses described below.

The questionnaires assessing outcome are all self-administered. The research associate (RA) made an appointment at baseline to meet with the patient again at 6 weeks, at which point the RA also gave the patient the second 6 week batch of medication. Those who did not attend this appointment were telephoned to offer a further appointment or to fill out the questionnaire by post if meeting was proving to be problematic. At the 6-week assessment an arrangement was made to meet again for the final time at 12 weeks.

It is expected that maximum treatment response to antidepressants would have occurred at 6 weeks (although the patients may continue to improve after that time). The 12-week follow-up is carried out as an indicator of a more persistent response to treatment. After exit from the trial, the general practitioner continues prescribing the medication for as long as clinically indicated.

### Outcome measures

#### Primary Outcome

The total BDI score at 6 weeks in a continuous form. This was chosen over a binary variable as use of the total BDI score should increase statistical power.

#### Secondary Outcomes

- Total BDI score at 12 weeks;

- proportion 'in remission' (defined as a total BDI score < 10) at 6 and 12 weeks;

- SF-12 at 6 and 12 weeks;

- HADS at 6 and 12 weeks;

- BFI-44 at 6 and 12 weeks;

- Side effects profile at 2, 6 and 12 weeks;

- Medication compliance at 2, 6 and 12 weeks.

### Statistical analysis

The analysis and reporting of this trial will be in full accordance with the CONSORT guidelines[25]. The participants in the trial will be compared with those eligible but not randomised using appropriate descriptive statistics and tests for means and proportions. Baseline comparability of the two randomisation groups will be ascertained using descriptive statistics. Moreover, the anticipated similarity of the two groups overall at follow-up will be verified using regression models to provide 95% confidence intervals of differences between the SSRI and NaRI groups in mean 6-week and 12-week BDI adjusting for baseline BDI and stratification. If necessary, similar analyses will be conducted for other secondary outcomes.

The primary comparative analyses for this trial are two pre-specified subgroup analyses for the primary outcome of BDI score at 6 weeks, with analysis performed on an intention-to-treat basis. Both of these will be investigated by introducing the interaction between treatment and the relevant binary predictor variable (genotype or severity) into a multiple regression model for the primary outcome (log transformed if appropriate), adjusting for baseline BDI score and stratification. The impact of missing values will be investigated in sensitivity analyses involving various assumptions and the use of substituted values.

Further secondary analyses will investigate alternative forms of the predictor variables. For instance, a separate category will be considered for those heterozygous for the insertion allele, as will continuous variables for severity (CIS-R). Other secondary analyses will also involve explanatory analyses such as models accounting for the degree of adherence with treatment. In particular, to complement the primary intention-to-treat comparison of the two groups, an 'explanatory' analysis will be conducted including only the 90% of participants who from early experience with the trial are continuing with their allocated medication at least three weeks after randomisation. As necessary, the above analyses will be repeated for all the secondary outcomes, using linear, logistic or repeated measures regression as necessary.

### Justification of the sample size

The sample size calculation for the GenPod trial employed a binary version of the primary outcome (BDI at 6 weeks) to facilitate both its formulation and interpretation. Specifically, the outcome was taken to be that of 'remission' (attaining a score of < 10 on BDI at 6 weeks). The transporter allele was also represented by a binary variable even though, in the absence of dominance or heterosis, heterozygotes would be expected to be intermediate in effect between the homozygotes. Both these approaches would be anticipated to be conservative in the sense of leading if anything to an underestimate of statistical power. In addition, the formulations all used a (2-sided) 5% significance level since although the two principal (subgroup) analyses involve the same outcome they are few in number (similar to the number of primary outcomes often involved in pragmatic trials) and reflect distinct hypotheses and types of predictor variables. Lastly, it was specified that remission would occur in 65% of participants (on average) in 6 weeks, regardless of the antidepressant used.

Given that the primary hypotheses related to interactions between 'exposures' on an outcome variable, the sample size was determined using a computer program for such situations available from the National Cancer Institute (NCI) based on the methods of Lubin and colleagues[26,27]. As would be expected in the context of interaction effects for binary variables, the sample size is large and very sensitive to the assumptions that are made[28,29]. Moreover, as is common for large-scale randomised trials in a community-based setting, recruitment to the GenPod trial was expected to be challenging, and in the event the accrual of patients into the trial in Bristol was even slower than anticipated. Hence as part of a request for extended funding (in addition to widening recruitment to a further two centres – Birmingham and Newcastle), a revised power calculation was presented. The description given here will therefore cover the original sample size calculation – in particular, the key specifications including (detectable) target effect sizes – and the implications of what currently is the likely final achieved sample size.

In relation to the transporter hypothesis, studies in the UK have observed 25% homozygous for the insertion allele and 50% heterozygous[30,30,31]. This accords with results from an Italian sample[13] and the frequency was therefore assumed to be 25%. In the original proposal it was stated that a substantial differential effect would be observed if the proportions in remission at 6 weeks were as follows: 80% among homozygotes for the insertion allele (l/l) on SSRIs, 65% in the NaRI group and 60% recovery for the remainder (s/l and s/s) on SSRIs and 65% recovery on NaRIs With 90% power it was calculated that this would require 754 subjects in total, increased to 887 to allow for 15% attrition at 6-weeks. This corresponds to an interaction odds ratio (θ) of 0.375. For the severity hypothesis, from previous data obtained from primary care by the research team, it was assumed in the original protocol that 44% would be in the more severely depressed group (≥ 28 on the CIS-R). With a target differential effect of 75% remission in the low severity/SSRI and high severity/NaRI groups compared with 55% remission in the other two groups, 282 participants would be needed for 90% power. The target sample size of 887 was therefore driven principally (and again conservatively) by the transporter hypothesis.

The extension request contained a range of revised likely sample sizes predicted to be available for analysis in both the intention-to-treat (ITT) and explanatory analyses; the resultant target for the ITT analyses was 676 followed up at 6 weeks, with lower power than originally (80% rather than 90%) but no reduction in sensitivity to the (transporter) interaction. The details given here have been further informed by the continuing (challenging) experience of recruitment to the trial, albeit with continuing high levels of follow-up – well over 90% at 6 weeks. Given the number randomised when the extension request was made, a final target of 570 for (primary) analysis was considered to be achievable, assuming the high follow-up rate continued.

We have also been able to estimate the actual remission rates in the trial and these were substantially less than the 65% expected. We have observed a 25% proportion with remission; therefore, the power calculations below have used this result.

The primary analysis proposes to use BDI score as a continuous outcome. One method of estimating the reduction in power is to compare the power for a main effect with the original sample size and the final target sample size of 570. Using Nquery, the power for a main effect was reduced by 9.1%. This calculation, of course, gives an approximate estimate of the reduction in power, if we assume that in broad terms, the reduction in power for the main effect will be similar to that for an interaction term. We also used the Lubin method (as above) and estimated that an interaction odds ratio (θ) of 0.33 could be detected with 80% power at the 5% level, based upon similar assumptions to those described above. This compares with the equivalent figures in the original power calculations of 90% power to detect a θ of 0.375, in turn translating to remission rates of 25% for NaRI and 36.8%/19.1% for homozygous/remainder in the SSRI group.

The figures presented here provide a realistic indication of the sensitivity of the GenPod trial to the primary interactions of interest. In addition, the detectable interactions retain the conservative aspects of the original specification: firstly, we have used a binary outcome rather than the continuous version that will be employed in the primary analysis; second, we focus on the transporter hypothesis for which power will be smaller; simplification of this exposure to binary homozygotes versus the remainder, whereas, the heterozygotes having an intermediate value is more in keeping with the likely biology. We have concluded that, given the uncertainties about the assumptions underpinning the power calculations, the shortfall in recruitment will have a relatively small impact on the ability of the trial to answer the primary research questions posed.

## Discussion

This randomised trial will address two important clinical questions. First, it will aid clinical decision-making and provide a rationale for targeting antidepressant treatment. It will allow GPs and psychiatrists to prescribe first line treatment with a higher prospect of effectiveness, thereby speeding recovery, and reducing the cost to the NHS. Pharmacogenetics is still widely viewed as a futuristic science. However, it is already possible in a research laboratory to conduct genotyping in less than 24 hours. Fully automated 'lab on a chip' technology will speed this process, reduce expense and improve availability. Predictive allele profiling could soon be a simple blood test[32,33].

The second advance is in improving our understanding of the biology of depression. At present, the different role of NA and 5HT in the psychopathology of depression is uncertain. Indeed, the study could contribute to the development of a clinically useful and biologically valid system for sub-typing depression.

To avoid statistical problems with multiple testing and to focus on the prior hypotheses posited, genotyping in the GenPod trial itself is being restricted to a single polymorphism. There are, however, many polymorphisms within other genes that may plausibly be involved in treatment response. DNA is therefore being extracted and banked to yield a major pharmacogenetics resource that will extend beyond the lifespan of this trial. In the future, this will facilitate provision of truly independent support or refutation of hypotheses generated by other researchers, or even enable the development of genome-wide SNP profiles predicting response[32].

## Current Study Status

The GenPod trial started recruiting participants in September 2005, and is due to complete data collection in March 2008.

## List of Abbreviations

5HT: 5-hydroxytryptamine – Serotonin; BDI: Beck Depression Inventory; BFI-44: Big Five Inventory; CIS-R: Clinical Interview Schedule-Revised; DNA: Deoxyribonucleic acid; HADS: Hospital Anxiety and Depression Scale; ITT: Intention-to-treat; MRC: Medical Research Council; NA: Noradrenaline; NaRI: Noradrenaline Reuptake Inhibitors; NCI: National Cancer Institute; NHS: National Health Service; NICE: National Institute of Clinical Excellence; PCTs: Primary Care Trusts; RA: Research Associate; RCT:  Randomised Controlled Trial; SF-12: A 12 Item Short Form Health Survey; SNRI: Serotonin Noradrenaline Reuptake Inhibitors; SSRI: Specific Serotonin Reuptake Inhibitors; TCA: Tricyclic Antidepressants.

## Competing interests

Reboxetine is manufactured by Pharmacia and the Citalopram used on this study was manufactured by Sterwin Medicines. Some of the authors have received fees from the pharmaceutical industry (listed below) although none of these fees were in relation to the medications used in this trial. PC is a paid member of advisory boards for Lilly, Sevier and Wyeth pharmaceutical companies. DN received lecture fees from Pharmacia, although not in regards to Reboxetine, and has a current consulting arrangement with Lundbeck. MO'D is the occasional recipient of fees for chairing symposia sponsored by pharmaceutical companies including Lilly, Janssen-Cilag and Astra-Zeneca. GL has also received occasional fees for speaking from the pharmaceutical industry. The remaining authors declare that they have no competing interests.

## Authors' contributions

GL, TP, DS, PC, DN, MO'D were responsible for the initial protocol, securing funding for the trial and the refinement of the protocol. JM, VM and NW contributed towards the refinement of protocol. JM is the trial co-ordinator responsible for the on-going management of the trial. DT and LT were responsible for the recruitment of patients to the study and the majority of the data collection. LT wrote the initial draft of the manuscript. All authors contributed to and approved the final manuscript.
